# Cobot Motion Planning Algorithm for Ensuring Human Safety Based on Behavioral Dynamics

**DOI:** 10.3390/s22124376

**Published:** 2022-06-09

**Authors:** Bo Liu, Weiping Fu, Wen Wang, Rui Li, Zhiqiang Gao, Lixia Peng, Huilong Du

**Affiliations:** 1 School of Mechanical and Precision Instrument Engineering, Xi’an University of Technology, Xi’an 710048, China; 1180211007@stu.xaut.edu.cn (B.L.); wangwen@xaut.edu.cn (W.W.); lirui@xaut.edu.cn (R.L.); gaozhiqiang@xaut.edu.cn (Z.G.); 1180211008@stu.xaut.edu.cn (L.P.); 2200220078@stu.xaut.edu.cn (H.D.); 2 School of Engineering, Xi’an International University, Xi’an 710077, China

**Keywords:** behavioral dynamics, collision avoidance, motion planning, psychological safety field model, safety in HRI

## Abstract

Recently, the safety of workers has gained increasing attention due to the applications of collaborative robots (cobot). However, there is no quantitative research on the impact of cobot behavior on humans’ psychological reactions, and these results are not applied to the cobot motion planning algorithms. Based on the concept of the gravity field, this paper proposes a model of the psychological safety field (PSF), designs a comprehensive experiment on different speeds and minimum distances when approaching the head, chest, and abdomen, and obtains the ordinary surface equation of psychological stress about speed and minimum distance by using data fitting. By combining social rules and PSF models, we improve the robot motion planning algorithm based on behavioral dynamics. The validation experiment results show that our proposed improved robot motion planning algorithm can effectively reduce psychological stress. Eighty-seven point one percent (87.1%) of the experimental participants think that robot motion planned by improved robot motion planning algorithms is more “friendly”, can effectively reduce psychological stress, and is more suitable for human–robot interaction scenarios.

## 1. Introduction

Recently, humans–robot collaboration (HRC) gained increasing attention from researchers and companies due to its higher flexibility and automation level without barriers. It is adequate to combine the workers’ flexibility with the power and accuracy of the robot [1,2,3]; it also can solve the problem of a companie’s transformation to multi-variety and small-batch production. However, an essential prerequisite is workers’ safety, for which researchers have carried out many research studies on [4], producing new safety standards relevant for HRC (ISO 10218 [5] and ISO/TS 15066 [6]). Moreover, some researchers have begun to pay attention on how to ensure the mental safety of workers in the process of HRC [7,8], how to improve workers’ acceptance and trust of robots, and the smoothness of completing assembly tasks. For example, in the literature [9], the authors state that the trust of human operators in their robot partners is key to the successful implementation of industrial HRC. In the article, it has been demonstrated that if a robotic agent is perceived by the operator to be unreliable or unpredictable it will increase the operator’s mental workload. In other words, if the operator does not have adequate trust in the robotic teammate, he is likely to place more mental resources on raw data to ensure that the robot is taking the correct actions, thus increasing the mental workload. During sustained periods of a taxing cognitive workload, humans typically display time-on-task (TOT) effects, in which performance becomes steadily worse over the period of task engagement [10].

Therefore, we believe that there is a great need to find a suitable robot motion planning algorithm so that the robot can work around the human operator with appropriate movements, reduce the cognitive load on the worker, and improve the operator’s comfort and trust in the robot. Therefore, our research is motivated to improve the robot motion planning algorithm so that the robot can achieve the goal of obstacle avoidance and reduce psychological stress. The objectives of our research are as follows:

1.  To quantitatively analyze the effect of the robot’s approaching human action elements on causing human psychological stress;
2.  To model the results of the quantitative study;
3.  To use the established psychological safety field model to improve behavioral dynamics so that the planned cobot motion trajectory can achieve the dual goals of obstacle avoidance and human psychological stress reduction.

This paper consists of nine sections. Section 2 briefly deals with state-of-the-art methods in ensuring physical and mental safety in the process of HRC. Section 3 summarizes the research methods used in this paper and the research process of this paper. Section 4 presents the collision detection and speed adjustment algorithms. Section 5 establishes the psychological stress field model. Section 6 describes the cobot motion planning algorithm of HRC based on behavioral dynamics. Section 7 describes the experimental procedure and the experimental results. Section 8 discusses the psychological safety field law experiment and the validation experiment of the cobot motion planning algorithm in this paper. Section 9 concludes the entire paper.

## 2. Related Work

Human safety is one of the biggest concerns in HRC. Previous works [11,12] focused on humans’ physical safety of HRC. Many methods are proposed for collision-free motion planning, such as probabilistic roadmap (PRM) and rapidly exploring random trees (RRT). These methods are usually combined with the concept of configuration space (C-space) to plan the collision-free path of a robot. ISO 10218 [5] proposes that its speed and distance are monitored when the human body moves around the robot or makes various actions. ISO/TS 15066 [6] proposed classifying HRC into four levels. At every level, different kinds of safety equations are developed and analyzed.

The four levels of HRC are specified in ISO/TS 15066, and the four levels are shown in Table 1. In levels 1, 2, and 3, the robot must have physical separation, such as a fence or distance monitoring to ensure a separation distance between the human and the robot. The robot stops when a person enters the fence or when the distance between the human and the robot is less than the specified separation distance. Such safety measures lead to inadequate HRC, limiting the smoothness of robot motion, and the robot stops once the worker enters the fence or if it is less than the specified separation distance, which sacrifices the robot’s efficiency. At level 4, although the physical safety of workers can be guaranteed to a certain extent, it does not ensure the psychological safety of workers.

However, this type of method significantly limits the smoothness of HRC and sacrifices the efficiency of collaboration greatly.

To solve this problem, many researchers improve the motion planning algorithm by studying the psychological impact of humans on robot motion in the process of HRC and propose perceived safety and human-aware motion planning algorithms to ensure physical HRC (pHRC) [2,13,14]. For example, Kulic et al. [15] and Zoghbi et al. [16] used PP (pick and place movement) and RR (reach and retrieve motion) tasks to simulate typical robotic tasks during HRC and captured the physiological responses of 36 participants during this process. The studies in these two articles collected skin conductance (SC) signals, heart rate signals, and EMG signals, combined with questionnaires and subjective survey data, to train HMM models to classify affective states and estimate arousal during collaboration. Kulic et al. [17] designed three robot speeds to enrich the inference model and proposed a feasible solution to this type of problem. However, this study only deals with human physiological responses in two movement modes, PP and RR, and does not address conditions such as distance or classify different body parts.

In [7], by collecting the participants’ SC signal and subjective score, it was concluded that workers will feel nervous when the robot approaches people, and it was suggested that the speed should be less than 0.5 m/s, the minimum distance should be greater than 2 m, and the robot should avoid linear movements. This type of research provides guiding suggestions for the exercise of robots, but the distance between cobots and workers is far less than 2 m. Such guiding tips are no longer suitable for more complex human–computer cooperative tasks.

Some studies [18,19,20] try to adjust the robot’s trajectory by predicting some limb positions of human beings in the future to reduce the conflict of shared space and improve human comfort. In [21], the researchers proposed a real-time safety system to measure the separation distance between the human and robot in real-time and realize accurate robot speed adjustment. In [22], based on the conclusions of existing literature, a trajectory planning method based on Variable Calculus was proposed to obtain a series of psychologically acceptable minimum acceleration trajectories to ensure people’s psychological safety during HRC. The advantage of this kind of method is that it can plan the trajectory and speed of the robot. However, the disadvantage is that it does not consider humans’ irrationality and psychological stress, which may affect a person’s actions. And this kind of method requires a larger space to reach the destination, making the robot’s arrival time longer.

It can be seen from previous research that the psychological responses caused by robots during HRC are a crucial factor in improving the quality of HRC [23], and it is also an essential basis for enhancing the robot motion planning algorithm. However, previous studies lack a systematic, complete survey on the psychological responses of robot speed, minimum distance, and different body parts. Therefore, we designed the psychological safety field law experiment, measured the psychological stress emotion generated by people when the robot approached different body parts at different speeds and minimum distances and established a psychological safety field model.

## 3. Methodology

**Figure 1:**
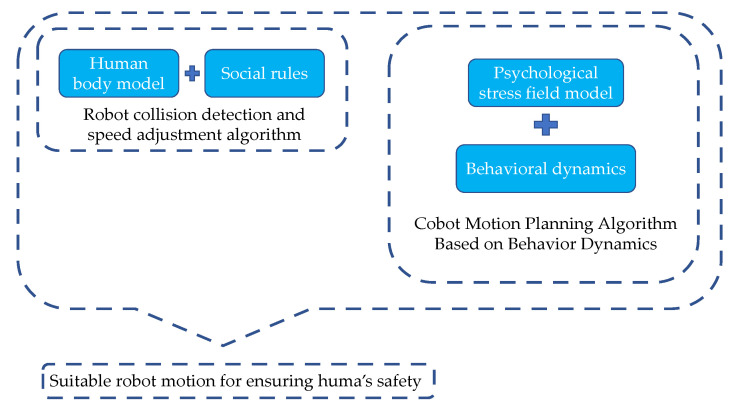
The schematic diagram of the research method.

In this section, we focus on describing our research methodology. Our ultimate goal is to design robot motion planning algorithms that can make the robot’s movements avoid the human body while reducing human psychological stress. To achieve this goal, first, we need to establish a collision detection and speed adjustment algorithm to detect the minimum distance between the upper and lower limbs of the operator and the robot in real-time and adjust the maximum speed of the robot according to the minimum distance. Secondly, we need to study the impact of the robot’s action factors on psychological pressure, derive the corresponding law, and mathematically model this law. Finally, we need to integrate the established collision detection and speed adjustment algorithm and the mathematical model of psychological stress into the behavioral dynamics motion planning algorithm. The integration will form a cobot motion planning algorithm that ensures human safety. The schematic diagram of the research method is shown in Figure 1.

To build collision detection and speed adjustment algorithms, we studied the related literature, and we found that people are very sensitive to the distance of others approaching them. For example, for friends and family, people usually allow each other to have access to intimate space, while, for colleagues, they may only allow each other to enter their personal space. Therefore, we modeled the human body and detected the distance between each part of the robot and the human model in real time. Then, considering the social rule factor, the robot’s maximum speed is specified when the distance between the robot and the human is less than the personal space and the intimate space, and the collision detection and speed adjustment algorithms are established.

In order to find the law of the effect of robot movement on human psychological stress and establish the psychological safety field model, we need to carry out an experimental analysis of three different factors, namely, different parts of the robot’s proximity to the human body, speed, and distance from the minimum separation, to find out the change law of psychological stress. We established the psychological safety field formula regarding the gravitational field formula and used the formula to express the psychological stress of human operators caused by static obstacles and robot movements.

In order to establish a robot motion planning algorithm that can guarantee the physical and psychological safety of human operators, the original behavioral dynamics motion planning algorithm needs to be improved. The already established psychological safety field formula has been added to the behavioral dynamics motion planning algorithm in a suitable manner to plan a suitable robot motion trajectory under the guidance of the real-time monitoring of minimum distance and maximum speed by collision detection and speed adjustment algorithms.

## 4. Robot Collision Detection and Speed Adjustment Algorithm

Social rules are the potential rules that guide HRC, and cobots can only assist humans with higher efficiency and acceptance in all its motion planning strategies to behave socially. This paper briefly introduces the human body model (HBM) and social rules. We propose collision detection and speed adjustment algorithms.

### 4.1. HBM and Social Rules

In the process of HRC, human position and motion are integrated into the robot motion planning algorithm, and the motion model of the robot is planned in terms of avoiding all parts of the human body. In order to reduce psychological stress, it is necessary to calculate the distance between each part of the human body and each node and the link of the robot and use the minimum distance as the basis to guide the robot away from the human body. Therefore, we will collect the joint bone points of the human body in the experiment to establish the human model.

**Figure 2:**
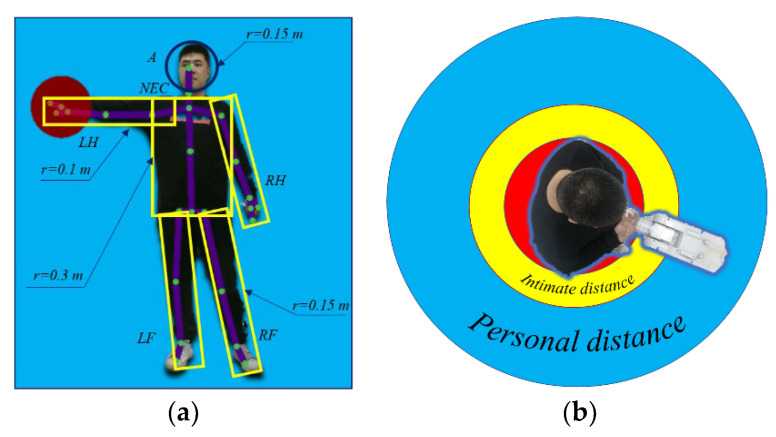
(**a**) Simplified topological structure model of a human body; (**b**) social rules model: the red circle represents the area of the human body, the yellow circle represents intimate distance, and the blue circle represents personal distance.

We collected the joint points of the worker’s head (A), two shoulders (LS and RS), two elbows (LE and RE), two hands (LH and RH), neck (NEC), spine (M), two hips (LH and RH), two knees (LN and RN), and two feet (LF, RF) by using a Kinect sensor. The topological model of the human body is as follows: The head is simplified as a sphere with a radius of 0.15 m; the upper body is simplified as a cylinder with a radius of 0.3 m; the axis of the cylinder is the line from NEC to M; the two arms are simplified as four cylinders with a radius of 0.1 m; the two legs are simplified as four cylinders with a radius of 0.15 m, as shown as Figure 2a.

How humans manage social space was first studied by Hall [24]; it suggests that humans are susceptible to the intrusion of other different Personal Spatial Zones [25]. The Personal Spatial Zones are divided into four distance zones, intimate distance, personal distance, public distance, and social distance, as shown in Figure 2b:

Intimate distance: ranges up to 0.45 m from the body, and interaction within this space might include physical contact.

Personal distance: ranges from 0.45 m to 1.2 m, which is used for interaction with friends.

The distance between the cobot and the human usually ranges from 1.5 m; thus, intimate distance and personal distance are the paper’s focus. 

When others enter the Personal Spatial Zones, they will generate psychological stress and discomfort in the social process. The distance between humans and a cobot is very close in the process of HRC. Cobots may cause discomfort or fears because of their inappropriate behavior [26]. To decrease psychological stress, humans may make a quick reaction, large-scale avoidance, and other risk avoidance actions, which will reduce work efficiency and even lead to physical injury.

### 4.2. Collision Detection and Speed Adjustment Algorithm for Extension and Abduction

Calculating the distance between the cobot and the extension of the upper body and the legs’ abduction is necessary to control the collision between the cobot and the human body. The moving speed of cobots toward humans should be adjusted based on social rules to decrease psychological stress in HRC. The GRB3016 robot used in the experiment does not have a collision sensing function; therefore, to ensure safety, the robot’s maximum speed in the investigation shall not exceed 0.25 m/s. According to the structure of the GRB3016 robot, it is only necessary to consider the 3rd, 4th, and 5th joints and the link *L_R_*_1_ between the 2nd and 3rd joints and the link *L_R_*_2_ between the 4th and 5th joints and the limbs of the human body.

The algorithm calculates the distance between the robot’s two links *L_R_*_1_ and *L_R_*_2_ and joints end effector, 2nd, 3rd, 4th, and 5th, and the simplified human body topology model finds the minimum distance *d*_min_ from each minimum distance and carries out motion planning and speed adjustment for the cobot. The collision avoidance and speed adjustment algorithm is shown in Algorithm 1.
**Algorithm 1** Collision avoidance and speed adjustment algorithms1.S←{stop, moving}; T←{stop, moving}2.t = 03.for end effector is moving do4.t = t + Δt5.${\vec{v}}_{\min}=0.01\;m/s$
6.if *d*_min_ ≤ The radius of the corresponding humans limb7.S.stop/*The robot stop */8.end if9.if the radius of the corresponding humans limb ≤ Dmin ≤ 0.4510.${\vec{v}}_{\max}=0.2\;m/s$/*The maximum velocity of the robot */11.end if12.if 0.45 ≤ *d*_min_ ≤ 1.213.${\vec{v}}_{\max}=0.25\;m/s$/*The maximum velocity of the robot */14.end if15.end if16.end for

## 5. Psychological Stress Field Model

To describe the psychological stress generated by humans when the robot approaches, we propose the concept of psychological safety field (PSF) by referring to the gravity field and the traffic safety field by Wang [27,28]. The definition of PSF is as follows: in the scene of HRC, humans are impacted by the static environment and the motion of cobots. We define that the psychological stress of avoiding static objects is called potential energy field $E_{I}$; due to the influence of cobot motion, humans have the psychological stress to avoid the moving cobot, which is called “kinetic energy field $E_{V}$”. The superposition of $E_{I}$ and $E_{V}$ generates PSF $E_{P}$, and PSF $E_{P}$ is shown in Formula (1).

(1) $$E_{P}=E_{I}+E_{V}$$

We establish the $E_{I}$ of $E_{P}$ by referring to the formula of the gravity field, which is mainly determined by the attribute conditions of static objects. The field strength of $E_{I}$ formed by the static objects at (*x_o_*, *y_o_*) is shown in Formula (2):

(2) $$E_{I}=-\frac{k_{o}\cdot q_{o}\cdot M_{o}}{d_{I}^{2}}d_{I}$$

where $q_{o}$ is determined by the nature, volume, and risk degree of static objects; $M_{o}$ is the mass of stationary objects; the unit is kg. $k_{o}$ is people’s cognition of static objects, the unit is N·m^2^/kg^2^, and the values are different according to work and life experience. $d_{I}$ and $d_{I}$ are the distance scalar and distance vector between the obstacle and the surrounding environment, respectively; the unit is m.

It can be seen from Formula (2) that $E_{I}$ has the attributes and dimensions of the physical field. By conducting calculations, the curl of $E_{I}$ is zero; that is, $curlE_{I}=\nabla \times E_{I}=0$, and it shows that $E_{I}$ is a conservative vector field (or an irrotational field). We define a potential energy function for the obstacle material point for a conservative vector field. The potential energy function is a scalar function about the potential vector, which is recorded as $SE_{I}$. $SE_{I}$. is the potential energy acting on human psychology by the conservative field force in $SE_{I}$, which has an energy dimension. When the distance between people and obstacles is infinite, we can obtain the potential energy field in the psychological security field-$SE_{I}$.

(3) $$SE_{I}=-\int_{\infty }^{d_{I}}F_{I}\cdot dd_{I}$$

Potential energy field force $F_{I}$ expression can be obtained from Equation (3). As shown in Equation (4), $F_{I}$ is $E_{I}$‘s negative gradient descent direction. $SE_{I}$ is positive and reflects the distribution of humans’ psychological stress caused by static objects in space. The greater $SE_{I}$ is, the greater the psychological stress.

(4) $$F_{I}=\frac{k_{o}\cdot q_{o}\cdot M_{o}}{d_{I}^{3}}d_{I}$$

On this basis, we define the “kinetic energy field” in the psychological safety field $SE_{V}$ related to cobots. This paper’s “kinetic energy” is not analogous to the concept with “kinetic energy” in physics. It is only used to describe the impact of cobot motion on human psychology to distinguish it from the effect of static objects on human psychology in the environment. $SE_{V}$ refers to the relevant definitions of $SE_{I}$. When the cobot is stationary, it is equivalent to another static object in the environment, and its psychological impact on people, i.e., $SE_{I}$ and $SE_{V}$, can also be calculated by Equation (3). However, when the cobot starts to move, the cobot’s motion can cause human psychological stress in different ways, and cobot motion changes the field strength of $SE_{I}$ in a “dynamic” linearly related manner; thus, $SE_{V}$ is the psychological stress caused by the speed and minimum distance between the nearest moving parts of cobot and human. Therefore, the final formulas of $SE_{V}$, “kinetic energy field” force-$F_{V}$***_,_*** and PSF can be expressed as follows:

(5) $$SE_{V}=-\frac{k_{r}\cdot q_{r}\cdot M_{r}\cdot Sp\left(d_{r},v\right)}{d_{V}^{2}}d_{V}$$

(6) $$F_{V}=-\frac{k_{r}\cdot q_{r}\cdot M_{r}\cdot Sp\left(d_{r},v\right)}{d_{V}^{3}}d_{V}$$

(7) $$SE_{P}=SE_{I}+SE_{V}$$

where $k_{r}$ is humans’ cognition of the danger degree of cobot motion, and the unit is N·m^2^/kg^2^, which depends on individual gender, personality, and experience. $q_{r}$ is the degree of danger of the cobot and its clamping objects, depending on its shape, volume, and clamping things. $M_{r}$ is the mass of the cobot, the unit is kg, and $d_{v}$ and $d_{v}$ are the scalar distance and vector distance between the center point of the cobot and the center point of the human body, respectively. $Sp\left(d_{r},v\right)$ is the influence law of different motion modes of the cobot on humans’ psychological stress. When the nearest moving part of a cobot to a person approaches the person at other minimum distance $d_{r}$, speed v, and approach direction, the psychological stress on the human body is different.

To obtain the influence law $Sp\left(d_{r},v\right)$ of psychological stress of when a cobot approach a person. It is necessary to study the influence law of the influence of each factor on psychological stress, which is challenging to describe directly by the theoretical model; thus, it is necessary to study the impact of each factor by conducting experiments. We obtained the psychological stress influence law $Sp\left(d_{r},v\right)$ caused by the speed and minimum distance of the robot approaching the human head, chest, and abdomen are obtained by conducting a comprehensive experiment.

**Figure 3:**
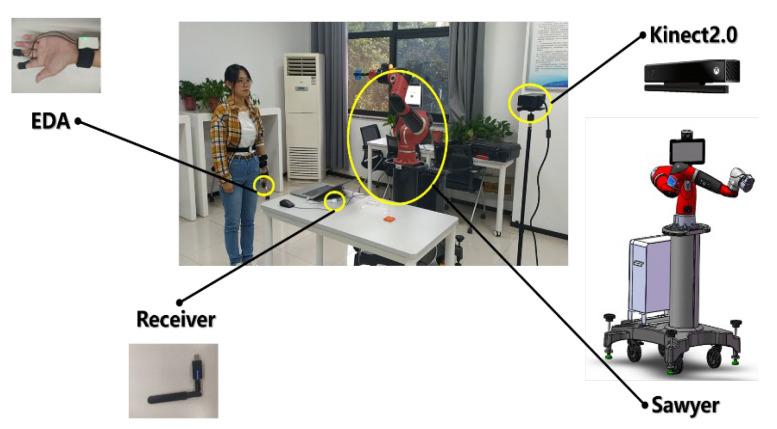
Test equipment and scenarios.

We designed the psychological safety field law experiment to determine the law of human psychological stress when the robot approaches the human body. Forty-one volunteers (29 males and 12 females) were recruited in the experiment as participants. We set a comprehensive experiment with the cobot speed of 0.5, 0.7, 0.9, and 1 m/s and the minimum distance of 0.1, 0.3, and 0.5 m. For example, the robot uses a speed of 0.5 m/s and a minimum spacing of 0.1 m approach to the participants’ motion. Firstly, Sawyer grabbed the building blocks from a fixed position on the table and approached the participants’ head at a constant speed of 0.5 m/s. The cobot stops at the minimum distance of 0.1 m from the participants’ face and holds the position for 10 s. Secondly, Sawyer returns to the same fixed height above the grasping position and holds the position for 10 s and then approaches the participants’ chest about 0.1 m at the same speed. Thirdly, Sawyer returns to the same fixed height above the grasping position and holds the position for 10 s and then approaches the participants’ abdomen about 0.1 m at the same speed. After the test, replace the cobot motion and complete the rest of the comprehensive experiment. During the experiment, the Kinect camera was used to collect the experiment scene, and the Electrodermal Activity (EDA) sensor was used to collect the Skin Conductance Response (SCR) generated by the participants when the cobot approached the participants. The experimental scenario is shown in Figure 3. The experimental scheme is shown in Table A1 in Appendix A.

Each participant sat quietly in the laboratory for 3 min before the experiment, excluding the psychological impact before entering the laboratory. In all experiments of each participant, we found the maximum SCR value of the event stimulus for each robot approaching as the reference value and divided the SRC value of each event by the maximum SCR value as the psychological stress response to the robot approaching operation, which is described in Equation (8):

(8) $$Sp=\frac{{\mathrm{SCR}}_{i,j}}{{\mathrm{SCR}}_{i,j\max}}$$

where *i* is the *i*th person (*I* = 1, 2, …, n), *j* represents the *j* experiment (*j* = 1, 2, …, n), and *p* is the close body parts (*p* = head, chest, abdomen).

After the experiment, count the mean value of the Sp value of all participants for all speeds and minimum distance of the head, chest, and abdomen; then, take the mean value of Sp value as the law of psychological stress of participants for cobot approaching motion and conduct surface fitting to obtain the law formula of psychological stress considering cobot motion. The law formulas for head, chest, and abdomen are Formulas (9)–(11), respectively. The parameters in Equations (9)–(11) are shown in Table A2 in Appendix A.

(9) $$Sh=z_{oh}+a_{h}\cdot d+b_{h}\cdot v+c_{h}\cdot d^{2}+d_{h}\cdot v^{2}+e_{h}\cdot d\cdot v$$

(10) $$Sc=z_{oc}+a_{c}\cdot d+b_{c}\cdot v+c_{c}\cdot d^{2}+d_{c}\cdot v^{2}+e_{c}\cdot d\cdot v$$

(11) $$Sa=z_{oa}+a_{a}\cdot d+b_{a}\cdot v+c_{a}\cdot d^{2}+d_{a}\cdot v^{2}+e_{a}\cdot d\cdot v$$

**Figure 4:**
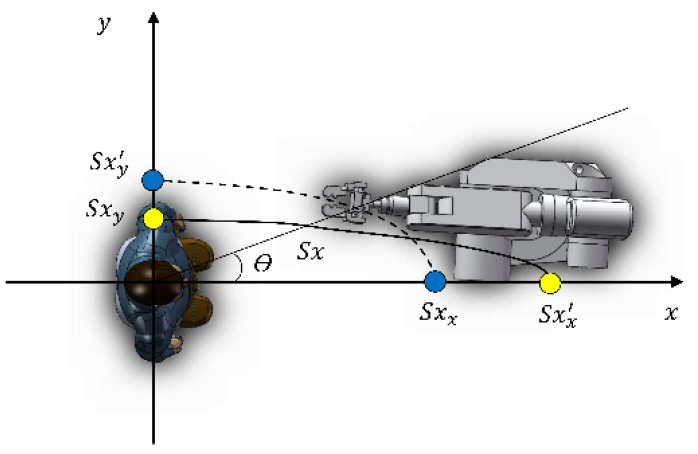
Schematic diagram of equipotential line.

Here, Sh, Sc*,* and Sa are the Sp values of the cobot approaching the head, chest, and abdomen, respectively. The parameters and variance of Formulas (9)–(11) are shown in Table 2. The final $SE_{I}$ equation is selected according to the part of the cobot closest to the human body; for example, if the chest is the most intimate part of the cobot as the cobot approaches the participants, Formula (10) should be chosen to form *SE_V_*. In the experiment, the participants can fully observe cobot motion, so the approach direction of the cobot cannot affect participants’ judgment of the motion state of the cobot. However, in actual work and life, humans’ attention cannot be fully focused on observing the cobot motion. Therefore, we believe that when the cobot approaches a person from the person’s side, the equipotential line of PSF becomes denser. Referring to the method of [21], when the cobot approaches from the front of the person, psychological stress reaches the maximum, and when it comes from the left or right side of the person, psychological stress reaches the minimum. The schematic diagram of the equipotential line is show in Figure 4.

$Sp_{x}$ is the corresponding Sp value at point *x* when the cobot approaches a specific part of the person from the front, and $Sp_{y}$ is the Sp value on the side of the person. At this time, $Sp_{x}=Sp_{y}$, and the form of the equipotential line is determined by human personality and cognition of the cobot. The corresponding mathematical expression of psychological stress is shown in Equation (12):

(12) $$\beta \left(\mu ,\theta \right)=Sp_{x}\left(d_{r},v\right)-\left(1-\mu \right)Sp_{x}\left(d_{r},v\right)\left|\sin\theta \right|$$

where ε is related to a person’s degree of concentration; the more you focus on your work, the smaller ε is. In this paper, we chose ε = 0.2; therefore, the final expression of $SE_{v}$ is as follows.

(13) $$\begin{array}{c} SE_{V}=-\frac{k_{r}\cdot q_{r}\cdot M_{r}}{d_{V}^{2}}\cdot \\ \ldots \left(Sp_{x}\left(d_{r},v\right)-\left(1-\mu \right)Sp_{x}\left(d_{r},v\right)\left|\sin\theta \right|\right)d_{V} \end{array}$$

The previous description does not give the determination methods of $k_{o}$, $q_{o}$, $k_{r}$*_,_* and $q_{o}$. For $k_{o}$, it can be determined by questionnaire or subjective evaluation, and the static environment can be scored using the psychological scale that can describe persons’ perception of static objects. On this basis, it can be divided by a constant to meet the individual’s psychological stress. For $q_{o}$, it can be obtained by scoring the shape sharpness of static objects, and this method can also be used to determine $q_{r}$. For $k_{r}$, it can be obtained by a similar subjective evaluation method or by measuring individual physiological signals as the cobot approaches the human body. For example, the cobot comes the head, chest, and abdomen of the human body at a speed of 1 m/s and a minimum distance of 0.1 m to obtain the Sp value, and the value of $k_{r}$ can be obtained by comparing it with Sh, Sc*,* and Sa obtained in the model, for example, $k_{r}=Sp/Sh$.

We randomly selected six participants to verify whether the psychological safety field model can express the psychological pressure and emotion caused by individuals. The experiment designed the cobot to approach the participants’ head, chest, and abdomen in a frontal approach at speeds of 0.9, 0.7, and 0.5 m/s respectively; the comprehensive experiment included minimum distances of 0.1, 0.3, and 0.5 m.

Compare the relative error between the experimental results and the values calculated by the empirical formula of Sp value and obtain the model’s accuracy. After the experiment, the relative errors of the head, chest, and abdomen were counted, and accuracy was calculated. Accuracy results are shown in Table 2. In this study, we only provided the main experiment and establishment process of the psychological safety field model, and the rest of our research results were published in Chinese journals.

## 6. Cobot Motion Planning Algorithm Based on Behavior Dynamics

### 6.1. The Feedback Principle of Cobot Motion Planning Based on Psychological Stress Field Model

**Figure 5:**
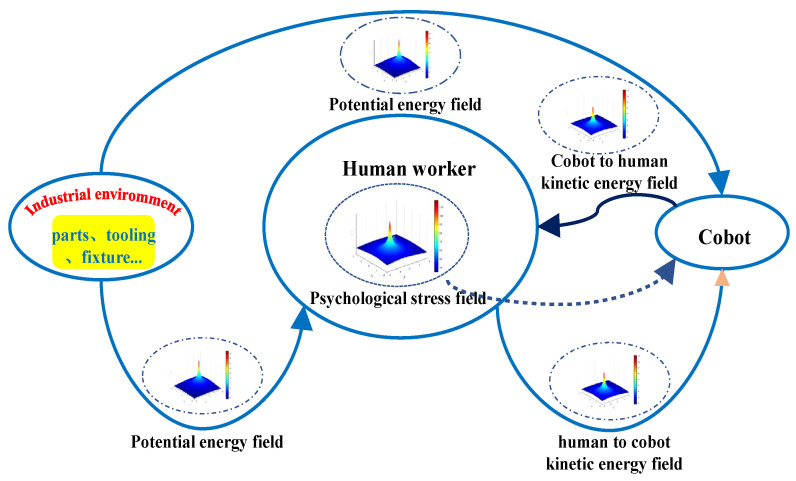
The feedback principle of cobot motion planning.

In 3D space, the basic principle of behavioral dynamics is as follows: In the cobot space coordinate system *XOYZ*, the cobot takes the target point as the attractor to generate attraction and the obstacle to be avoided as the repulsor to generate repulsion. The combined action of the two produces the optimal trajectory of the cobot to reach the target point. In behavioral dynamics, the cobot’s behavior toward the target point is regarded as “attraction”, and the behavior of avoiding obstacles is viewed as “repulsion force”. Although the “attraction/repulsion force” are virtual forces, they have the dimension of force in physics. $F_{I}$ is established in PSF, and $F_{V}$ has the same dimension of force; the two kinds of forces can be added directly. Therefore, we introduce the human PSF model as feedback into the motion planning algorithm as the basis of cobot motion planning. When the cobot moves toward the target by the “attraction” and “repulsion” of the behavioral dynamics motion planning algorithm, if there are humans in the environment, $F_{I}$ and $F_{V}$ shall be superimposed at the same time; that is, the cobot is comprehensively affected by the superposition of physical field and PSF, and its feedback principle is shown in Figure 5.

### 6.2. The Cobot Motion Planning Based on Behavioral Dynamics

**Figure 6:**
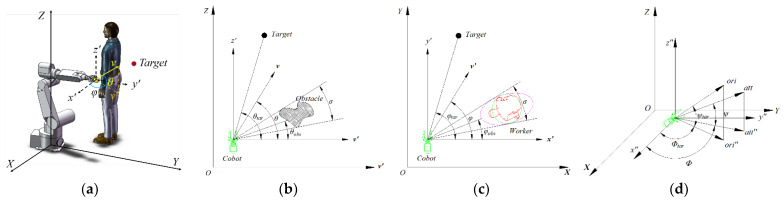
(**a**) Schematic diagram of the Cartesian coordinate system, local coordinate system, and auxiliary coordinate system; (**b**) the projection of *v′OZ*′ plane of end effector; (**c**) the projection of XOY plane of end effector; (**d**) the current posture of end effector and target posture.

Taking the base of the cobot as the origin, the Cartesian coordinate system *XOYZ* is established, and the local coordinate system *x*′*o*′*y*′*z*′ and auxiliary coordinate system *x*″*o*″*y*″*z*″ of the end effector are set with the center point of the end effector of the cobot as the origin. The axes of the three coordinate systems are the same, as shown in Figure 6a,d.

First, the target-oriented behavior is designed, and the end effector sets the velocity equilibrium point of the target-oriented point to a suitable finite value. When the weight of the target-oriented behavior is activated, velocity $\vec{v}$ tends to the desired velocity gradually and stably with the dynamic system. The target-oriented behavior model of the cobot is as follows:

(14) $$\left\{\begin{array}{l} \dot{\theta }=f_{tar}\left(\theta \right)=-\lambda_{tar}\left(\theta -\theta_{tar}\right)\left(-e^{-c_{1}d_{tar}}+c_{2}\right)\\ \dot{\varphi }=g_{tar}\left(\varphi \right)=-\lambda_{tar}\left(\varphi -\varphi_{tar}\right)\left(-e^{-c_{1}d_{tar}}+c_{2}\right)\\ \dot{v}=h_{tar}\left(\theta \right)=-\lambda_{v}\left(v-v_{\max}\left(1-e^{-cvd_{tar}}\right)\right) \end{array}\right.$$

where $\lambda_{tar}$ and $\lambda_{v}$ are the attraction strength of the dynamic system, which affects the convergence rate of the dynamic system, $d_{tar}$ is the distance from the target to the end effector, and $c_{1}$ is the coefficient of the attraction formed by the location of the target varying with the distance $d_{tar}$; $c_{2}$ is the global minimum attraction intensity; $c_{v}$ is the influence coefficient of velocity change. Taking $\vec{v}$, *φ*, and *θ* as behavior variables, as shown in Figure 6b,c, the yaw angle *φ* and pitch angle *θ* related to the velocity of the end-effector are calculated to guide the cobot to move to the target.

Secondly, in the design of avoiding human behavior, the cobot needs to avoid the extension and abduction of the person and other large movements. We consider the requirements of psychological stress for cobot obstacle avoidance and use $F_{I}$ and $F_{V}$ generated by a person for feedback regulation. The “repulsive force” of avoiding obstacles in the physical field and the $F_{V}$ of psychological safety are added. When the cobot avoids obstacles previously, at the same time, the cobot avoids the human body at a certain angle and speed. The behavior dynamics model of the avoidance behavior of the end effector is designed, as shown in Formula (15).

(15) $$\left\{\begin{array}{c} \dot{\varphi }=g_{obs}\left(\varphi \right)=-\lambda_{obs}\left(\varphi -\varphi_{obs}\right)e^{-c_{3}d_{i}}e^{c_{4}v}e^{-\frac{\left(\varphi -\varphi_{obs}\right)}{2\sigma^{2}}}+\\ \;\;\;\;\;\ldots c_{5}\left(F_{I}+F_{V}\right)\left(\varphi -\varphi_{obs}\right)\\ \dot{v}=h_{obs}\left(v\right)=-\left(\lambda_{v}+c_{6}\left(F_{I}+F_{V}\right)\right)\cdot \\ \;\;\;\;\;\ldots \sin\varphi \left[v-\min\left[\max\left[v_{\min},R\dot{\varphi }\right],v_{\max i}\right]\right] \end{array}\right.$$

Thirdly, the pose of the end effecter attitude-behavior model’s design is shown in Figure 6d. The orientation line of the target is *ori_ta_*. The projection of its auxiliary coordinate system *x*″*o*″*y*″-plane is *ori*″*_tar_*. The included angle between *ori_tar_* and *ori*″*_tar_* is *φ_tar_*, and the included angle between *ori*″*_tar_* and *x*″-axis is *ψ_tar_*. The orientation line of the end effector is *ori_ed_*. The projection of its local coordinate system *x*″*o*″*y*″-plane is *ori*″*_ed_*. The angle between *ori_ed_* and *ori*″*_ed_* is *φ_ed_*, and the angle between *ori*″*_ed_* and *x*″-axis is *ψ_ed_*. Taking *φ_ed_* and *ψ_ed_* as behavior variables, the posture-behavior model of the end effector is shown in (16):

(16) $$\left\{\begin{array}{l} \dot{\theta }=f_{ori}\left(\varphi \right)=-\lambda_{ori}\left(\phi -\phi_{tar}\right)\\ \dot{\psi }=g_{ori}\left(\psi \right)=-\lambda_{ori}\left(\psi -\psi_{tar}\right) \end{array}\right.$$

where $\lambda_{ori}$ is the attraction strength of cobot posture motion, and $\lambda_{ori}$ is used to adjust the convergence rate of the model.

Finally, the overall behavior model is obtained after linear superposition by designing the weight coefficient, which changes dynamically with the system’s relative position and speed factors, as shown in (17). We obtained a robot motion planning algorithm based on behavioral dynamics, namely Equation (17). Using Equation (17), the trajectory and speed of the robot can be provided in real time to provide the target point and speed at the next moment until it finally reaches the target:

(17) $$\left\{\begin{array}{l} \ddot{\varphi }=b\dot{\varphi }+\left|\omega_{tar}\right|g_{tar}\left(\varphi \right)+\left|\omega_{obs}\right|g_{obs}\left(\varphi \right)\\ \ddot{\theta }=b\dot{\theta }+\left|\omega_{tar}\right|f_{tar}\left(\theta \right)\\ \dot{v}=\left|\omega_{tar}\right|h_{tar}\left(\varphi \right)+\left|\omega_{obs}\right|h_{obs}\left(\varphi \right) \end{array}\right.$$

where *ω_tar_* ∈ [0, 1] and *ω_obs_* ∈ [0, 1] are the weights of the end effector’s tendency target and obstacle avoidance behavior, and *b* is the damping coefficient of the dynamic system to prevent the trajectory from diverging. The parameters are obtained by referring to [28] and making adjustments across many experiments. The parameters are shown in Table 3.

## 7. Experiment and Results

### 7.1. The Cobot Motion Planning Based on Behavioral Dynamics

**Figure 7:**
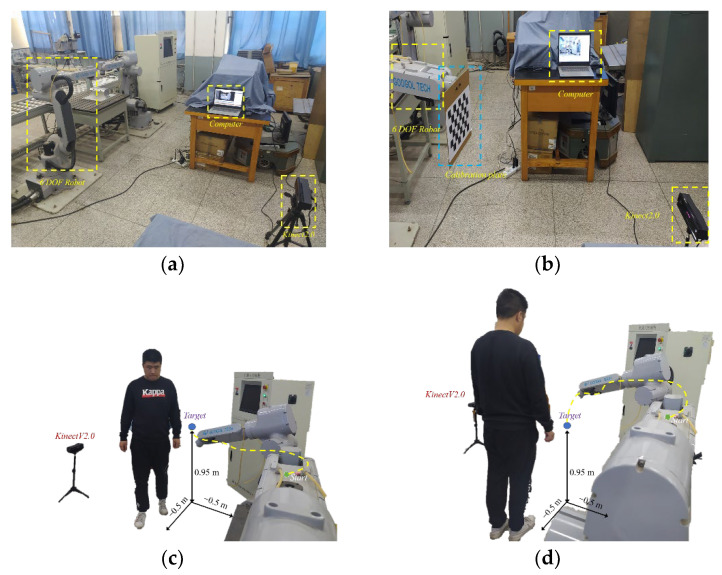
(**a**) The experimental equipment; (**b**) the calibration experiment; (**c**) the first experiment; (**d**) the second experiment.

In this section, experiments are designed to test the system’s performance. Two experiments are designed based on the GRB3016 robot, a 6-DOF light robot. The experimental equipment consists of a computer, a GRB3016 robot, and a Kinect sensor; the experiment scene is shown in Figure 7a. Two experiments are shown in Figure 7c,d.

To transfer the collected data of joint points of the experiment to the coordinate system XOYZ, we calibrate the camera and cobot. Firstly, the calibration board is combined with the end effector to collect pictures. Then, the internal and external parameters of the RGB camera and the infrared camera of Kinect v2.0 are calculated by using the camera calibration toolbox in MATLAB. Finally, the coordinate data of joint points of the experimenter are converted into the coordinate system of the cobot (that is, XOYZ). The calibration site is shown in Figure 7b; the transformed coordinate data of joint points are sent to the computer in real time. The robot’s trajectory is adjusted in real time according to the motion planning algorithm.

Three experimenters of our group participated in the two experiments, and all three experimenters were not familiar with the GRB3016 robot. During two experiments, experimenters moved aimlessly and repeatedly near the robot target to verify whether the PSF model can reduce psychological stress.

Experimental procedure:

Two experiments are designed in this paper, as shown in Figure 7c,d. In experiment 1, we use the motion planning algorithm without improved behavioral dynamics. In Experiment 2, we use the cobot motion planning algorithm to ensure human safety based on the behavioral dynamics proposed in this paper.

Before the experiment, we let the experimental participants sit still for 5 min to exclude external stimuli. After the experiment officially started, the participants stood still at the starting point for 25 s, after which the robot and the participant started moving at the same time. At this point, we asked participants to move from the start to the end several times until the robot reached the target. During the experiment, participants were told not to avoid the robot deliberately. This was performed to bring the participants as close to the robot as possible and to verify whether the improved motion planning algorithm proposed in this paper could reduce participants’ psychological stress. We collected the participant’s corrugator supercilia muscle (CS) EMG during the experiment using the ErgoLAB 3.0 cloud platform of Kingfar Technologies, Inc. The participants were involved in two experiments, one of which the robot’s motion trajectory was planned by the improved motion planning algorithm proposed in this paper and the unimproved motion planning algorithm planned by the other. The order of the two experiments was randomized. After the experiment, participants were asked to evaluate SAM for both experiments based on their own judgment and received an interview.

The interview questions are as follows:

1. Which robot movements do you think are more “friendly” and reduced your psychological stress?
2. If you think the motion planning algorithm that takes psychological stress into account is better at reducing your psychological stress, which robot movements make you feel that way?
3. Your suggestions for the robot.

### 7.2. Result

We analyzed SAM data after the experiment, and the mean values of participants’ ratings of both valance and arousal for both experiments are shown in Table 4. As shown in Table 4, our proposed improved algorithm scored greater in valance than the unimproved algorithm and less in arousal than the unimproved algorithm. The results indicate that the participants subjectively perceived that the improved motion planning algorithm reduced the participant’s psychological stress to a great extent.

**Figure 8:**
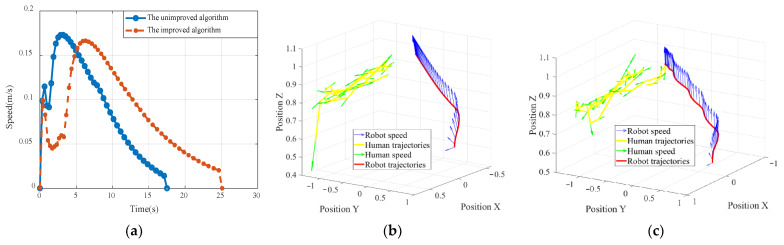
(**a**) Robot speed curve with the unimproved algorithm (blue) and robot speed curve with the improved algorithm (red); (**b**) speed and trajectory of the robot and participant in the experiment with the unimproved algorithm; (**c**) speed and trajectory of the robot and participant in the experiment with the improved algorithm.

To more visually compare the results of the two experiments, we analyzed one of the participants as an example, as shown in Figure 8. In the experiment with the unimproved robot planning algorithm, the robot did not avoid the participant when the participant approached the robot. This is because the unimproved algorithm can complete the avoidance action, but it does not consider the psychological safety factor and cannot adjust its trajectory and speed according to the psychological pressure of the participant. The robot’s trajectory and speed are shown in Figure 8a,b. In the experiments with the improved motion planning algorithm, we found that the robot could automatically adjust its motion trajectory when it encountered participants approaching the robot, avoiding the approaching participants with a larger avoidance angle and reducing the speed in an attempt to reduce the psychological stress caused relative to the participants; the robot’s trajectory and speed are shown in Figure 8a,c.

**Figure 9:**
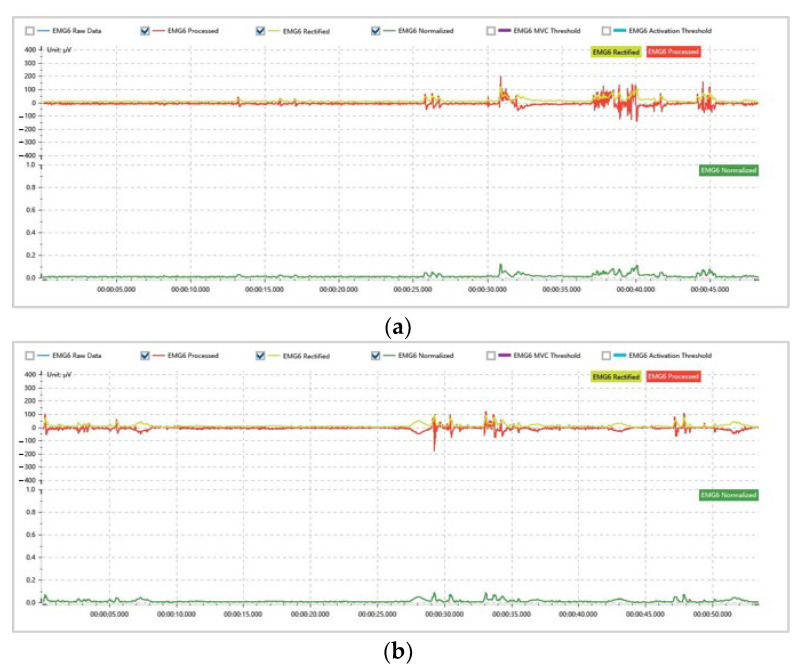
(**a**) EMG signal of the participant in the experiment with the unimproved algorithm; (**b**) EMG signal of the participant in the experiment with the improved algorithm.

We analyzed the physiological signals of the participants during the experiment. The EMG signals were normalized by wavelet noise reduction and moving RMS filtering using a window size of 100 ms. The obtained results are shown in Figure 9.

The time-domain analysis of the EMG signals was performed, and the results are shown in Table 5 and Table 6. Table 5 and Table 6 show nine characteristics of the EMG signals generated by the participants in the two experiments. We found that in both Processed EMG and Rectified EMG signals, the data for the mean and maximum values of EMG, standard deviation, variance, root mean square (RMS), mean absolute value, and integrated EMG (iEMG) values of the participants in the experiment with the improved motion planning algorithm were smaller than those in the experiment without the improved motion planning algorithm. Moreover, the longer length of the data using the improved motion planning algorithm illustrates the lower average speed of the robot.

After all participants completed the experiment, we statistically analyzed the Rectified EMG signal data of all participants and calculated the mean values of all relevant features, and the results are shown in Table 7.

The comparison revealed that our proposed improved motion planning algorithm’s mean, standard deviation, and variance were smaller than those of the unimproved motion planning algorithm. However, a one-way ANOVA of the EMG signals caused by the two algorithms is still needed to determine whether there is a significant difference in the psychological stress caused by different motion planning. We analyzed the mean values of Rectified EMG signals, and the psychological stress caused by both algorithms was significant (F(1,29) = 4.7 > Fα = 0.05(1,29) = 4.183, *p* < 0.05) with a confidence level of 96.59%. The results of the one-way ANOVA verified that the robot motion planned by the improved motion planning algorithm was indeed effective in reducing participants’ psychological stress.

The interview questions showed that for question 1, 87.1% of the participants thought that the improved motion planning was more friendly than the unimproved motion planning, 9.7% believed that the two algorithms were comparable, and 3.2% believed that the unimproved motion planning algorithm was better. For question 2, most participants believed that the robot could avoid the participant at a greater angle when approaching the robot and subjectively believed that the robot was “polite” and that such behavior would be less psychologically stressful. For question 3, some participants suggested the possibility of adding a voice dialogue function to the robot to prompt the participants before avoiding them.

## 8. Discussion

We would like to illustrate and discuss the limitations of the psychological safety field law experiment and the validation experiment of the robot motion planning algorithm in this paper. The main limitation in the psychological safety field law experiment was that the personality of the experimental participants was not considered in the experiment. The age range of the experimental participants was concentrated between 20 and 30 years old, and there were no experimental participants of older ages, which also made the psychological safety field model less accurate. To eliminate such an effect, we set parameter $k_{r}$ in the psychological safety field model equation, i.e., Equation (13), in order to accommodate different personalities, ages, and people with different experiences.

In the verification experiments of the robot motion planning algorithm, the main limitation of the robot motion planning algorithm proposed in this paper is that it was constrained by experimental conditions; our experiments were conducted in a quiet laboratory environment and not in a factory environment, where human psychological stress relative to robot movements may be different in a factory environment with workload and noise. To eliminate this effect, $\lambda_{tar}$ and $\lambda_{v}$ can be adjusted to improve the effectiveness of the robot motion planning algorithm in a factory environment.

## 9. Conclusions

This paper proposed an improved cobot motion planning algorithm to decrease psychological stress when workers collaborate with cobots in the scene of HRC. The main contributions of this paper are as follows:

1.  A collision detection and speed adjustment algorithm was established.
2.  We designed the psychological safety field law experiment, measured the psychological stress when the cobot approached different body parts at different speeds and minimum distances, and established a psychological safety field model.
3.  We improved the robot motion planning algorithm based on behavioral dynamics with the established psychological safety field (PSF) model as feedback.

We designed a validation experiment to verify whether the improved cobot motion planning algorithm can effectively reduce the psychological stress of participants. The results of the validation experiments show that the improved robot motion planning algorithm can effectively reduce human psychological stress and is more suitable for human–robot collaboration scenarios. Our future research focuses on how to adjust the motion algorithm to fit populations with different personalities.

## Figures and Tables

**Table 1:** Type of collaboration operation.

ISO/TS 15066	Type of Cobot Operation	Main Means of Risk Reduction
The first level	Safety-rated monitored stop	The robot stops when the worker enters the fence.
The second level	Hand guiding	The robot’s movements can only be guided manually by a worker.
The third level	Speed and separation monitoring	When the separation distance is less than the minimum separation distance, the robot stops.
The fourth level	Power and force limiting by inherent design or controls	Robot action stops when a human and robot collide.

**Table 2:** Analysis of Accuracy of participants’ ${S_{p}}^{\prime }$.

Participants	1	2	3	4	5	6
kr	1.34	1.73	1.41	0.81	0.84	0.85
head	83%	75.5%	78.8%	74.8%	67.2%	84.4%
chest	86%	65%	70.2%	65%	63.9%	75.5%
abdomen	85.1%	66.6%	69.1%	68.2%	68.7%	81.3%

**Table 3:** The parameters.

The Weight of the End Effector’s Behavior				The Initial Parameters			
*λ_tar_*	0.2	*ω_tar_*	0.9	*θ*	0.1	*c*_2_	0.5
*λ_obs_*	0.5	*ω_obs_*	0.1	*φ*	0.1	*c*_3_	0.15
*λ_v_*	0.3	*k*_1_	1.2	*φ*	0	*c*_4_	0.01
*λ_ori_*	0.2	*k*_2_	0.3	*ψ*	0	*c*_5_	0.00025
*b*	2	*p_a_*	0.02	*v*	1	*c*_6_	0.0002
*α_soc_*	0.4	*p_b_*	50	*c*_1_	0.1		

**Table 4:** The mean values of participants’ ratings of both valance and arousal.

SAM		
	Mean (Valence)	Mean (Arousal)
The improved algorithm	6.2	7.5
The unimproved algorithm	3.8	6.5

**Table 5:** Time-domain analysis of EMG signals of the participant in experiments with unimproved algorithms.

Data	Mean (μV)	Max (μV)	Min (μV)	Std (μV)	Variance (μV²)	RMS (μV)	Mean Absolute Value (μV)	iEMG (μV)	Data Length
Processed EMG	−6.85	199.45	−147.6	19.57	383.1	20.74	13.71	662.74	98,979
Rectified EMG	14.76	122.41	2.07	14.56	212.07	20.74	14.76	713.57	98,979
Normalized EMG	0.01	0.12	0	0.01	0	0.02	0.01	0.71	98,979

**Table 6:** Time-domain analysis of EMG signals of the participant in experiments with improved algorithms.

Data	Mean (μV)	Max (μV)	Min (μV)	Std (μV)	Variance (μV²)	RMS (μV)	Mean Absolute Value (μV)	iEMG (μV)	Data Length
Processed EMG	−7.17	118.58	−180.94	15.59	242.99	17.16	11.07	591.96	109,564
Rectified EMG	12.01	88.79	0.99	12.25	150.01	17.16	12.01	642.75	109,564
Normalized EMG	0.01	0.09	0	0.01	0	0.02	0.01	0.64	109,564

**Table 7:** Time-domain analysis of EMG signals for all participants.

Data	Mean (μV)	Max (μV)	Std (μV)	Variance (μV²)	RMS (μV)	Mean Absolute Value (μV)	iEMG (μV)
The unimproved algorithm	16.87	128.46	20.12	382.98	21.08	16.87	730.66
The improved algorithm	10.92	91.07	11.86	140.66	16.87	10.92	631.16

## Appendix A

**Table A1:** Experimental scheme.

Experimental Scheme	Body Parts	Minimum Distance (m)	Speed (m/s)
1	Head	0.1	0.5
	Chest	0.3	0.5
	Abdomen	0.5	0.5
2	Head	0.3	0.7
	Chest	0.5	0.7
	Abdomen	0.1	0.7
3	Head	0.5	0.9
	Chest	0.1	0.9
	Abdomen	0.3	0.9
4	Head	0.3	0.5
	Chest	0.5	0.5
	Abdomen	0.1	0.5
5	Head	0.5	0.7
	Chest	0.1	0.7
	Abdomen	0.3	0.7
6	Head	0.1	0.9
	Chest	0.3	0.9
	Abdomen	0.5	0.9
7	Head	0.5	0.5
	Chest	0.1	0.5
	Abdomen	0.3	0.5
8	Head	0.1	0.7
	Chest	0.3	0.7
	Abdomen	0.5	0.7
9	Head	0.3	0.9
	Chest	0.5	0.9
	Abdomen	0.1	0.9

**Table A2:** The parameters in Equations (9)–(11).

(a)		
$Sh=z_{oh}+a_{h}\cdot d+b_{h}\cdot v+c_{h}\cdot d^{2}+d_{h}\cdot v^{2}+e_{h}\cdot d\cdot v$		
parameter	Value	Standard Error
*z*_0*h*_	0.46965	0.10049
*a_h_*	−2.2376	0.38261
*b_h_*	−0.16461	0.32712
*c_h_*	2.85319	0.52145
*d_h_*	0.44897	0.25018
*e_h_*	0.12317	0.29329
R-Square	94.09%	
(**b**)		
$Sc=z_{oc}+a_{c}\cdot d+b_{c}\cdot v+c_{c}\cdot d^{2}+d_{c}\cdot v^{2}+e_{c}\cdot d\cdot v$		
parameter	Value	Standard Error
*z*_0*c*_	0.20524	0.02471
*a_c_*	−0.03063	0.09409
*b_c_*	−0.115	0.08045
*c_c_*	0.0126	0.12824
*d_c_*	0.18031	0.06153
*e_c_*	−0.10197	0.07213
R-Square	92.54%	
(**c**)		
$Sa=z_{oa}+a_{a}\cdot d+b_{a}\cdot v+c_{a}\cdot d^{2}+d_{a}\cdot v^{2}+e_{a}\cdot d\cdot v$		
parameter	Value	Standard Error
*z*_0*a*_	0.0914	0.04336
*a_a_*	−0.47717	0.1651
*b_a_*	0.31549	0.14116
*c_a_*	0.4543	0.22501
*d_a_*	−0.13909	0.10796
*e_a_*	0.15166	0.12656
R-Square	85.99%	
